# A randomized cross-over study of the quality of cardiopulmonary resuscitation among females performing 30:2 and hands-only cardiopulmonary resuscitation

**DOI:** 10.1186/1472-6955-8-6

**Published:** 2009-07-07

**Authors:** Cynthia Trowbridge, Jesal N Parekh, Mark D Ricard, Jerald Potts, W Clive Patrickson, Carolyn L Cason

**Affiliations:** 1 Department of Kinesiology, University of Texas at Arlington, Arlington, Texas, USA; 2 Human Neuromechanics Lab, Department of Kinesiology, University of Michigan, Ann Arbor, Michigan, USA; 3 ECC Research, Development and Innovation, American Heart Association, Dallas, Texas, USA; 4 Laerdal Americas, Laerdal Medical Corporation, Wappingers Falls, New York, USA; 5 School of Nursing, University of Texas at Arlington, Arlington, Texas, USA

## Abstract

**Background:**

Hands-Only cardiopulmonary resuscitation (CPR) is recommended for use on adult victims of witnessed out-of-hospital (OOH) sudden cardiac arrest or in instances where rescuers cannot perform ventilations while maintaining minimally interrupted quality compressions. Promotion of Hands-Only CPR should improve the incidence of bystander CPR and, subsequently, survival from OOH cardiac arrest; but, little is known about a rescuer's ability to deliver continuous chest compressions of adequate rate and depth for periods typical of emergency services response time. This study evaluated chest compression rate and depth as subjects performed Hands-Only CPR for 10 minutes. For comparison purposes, each also performed chest compressions with ventilations (30:2) CPR. It also evaluated fatigue and changes in body biomechanics associated with each type of CPR.

**Methods:**

Twenty healthy female volunteers certified in basic life support performed Hands-Only CPR and 30:2 CPR on a manikin. A mixed model repeated measures cross-over design evaluated chest compression rate and depth, changes in fatigue (chest compression force, perceived exertion, and blood lactate level), and changes in electromyography and joint kinetics and kinematics.

**Results:**

All subjects completed 10 minutes of 30:2 CPR; but, only 17 completed 10 minutes of Hands-Only CPR. Rate, average depth, percentage at least 38 millimeters deep, and force of compressions were significantly lower in Hands-Only CPR than in 30:2 CPR. Rates were maintained; but, compression depth and force declined significantly from beginning to end CPR with most decrement occurring in the first two minutes. Perceived effort and joint torque changes were significantly greater in Hands-Only CPR. Performance was not influenced by age.

**Conclusion:**

Hands-Only CPR required greater effort and was harder to sustain than 30:2 CPR. It is not known whether the observed greater decrement in chest compression depth associated with Hands-Only CPR would offset the potential physiological benefit of having fewer interruptions in compressions during an actual resuscitation. The dramatic decrease in compression depth in the first two minutes reinforces current recommendations that rescuers take turns performing compressions, switching every two minutes or less. Further study is recommended to determine the impact of real-time feedback and dispatcher coaching on rescuer performance.

## Background

To enhance survival of adult victims of cardiac arrest, the American Heart Association (AHA) Guidelines for Cardiopulmonary Resuscitation and Emergency Cardiovascular Care, updated in 2005, recommend that rescuers deliver cardiopulmonary resuscitation (CPR) in cycles of 30 chest compressions and 2 ventilations (30:2 CPR) at a rate of 100 compressions per minute with a compression depth of 1½ to 2 inches (38 to 51 millimetres (mm))[1] If multiple rescuers are present, the guidelines recommend that rescuers take turns compressing the chest to help reduce fatigue, changing rescuer every 2 minutes. The 2005 Guidelines and a subsequent AHA Advisory [2] focus on providing good quality chest compressions with minimal interruptions. To that end, use of Hands-Only (compressions-only) CPR is recommended when bystanders are providing CPR to an adult victim of out-of-hospital (OOH) witnessed cardiac arrest or when a bystander is not able to combine ventilations with chest compressions with minimal interruptions. It is also recommended that emergency medical dispatchers describe Hands-Only CPR when giving instructions over the phone to bystanders at the scene of a probable adult sudden cardiac arrest[1,3]

Hands-Only CPR has the potential for improving the chance of survival from OOH cardiac arrest by reducing the time to initiation of chest compressions and limiting interruptions in compressions associated with ventilations, resulting in a greater number of chest compression during the first few minutes after cardiac arrest[2] The AHA's 2008 Science Advisory acknowledges that further study is needed to assess the bystander's ability to deliver continuous chest compressions of adequate rate and depth for prolonged durations as might be required of a single bystander until further help arrives.

In training settings where compressions and ventilations CPR is performed on manikins, both lay providers and health professionals are able to deliver compressions at the recommended rate of approximately 100 per minute when CPR is delivered for up to 15 minutes[4,5] The preponderance of evidence, however, indicates that compressions of adequate depth decline rapidly after the first minute of CPR[4,5] but that rescuers do not recognize the performance deterioration until almost 2 minutes after compression depth has become inadequate[6].

Studies of rescuer ability to maintain effective chest compression rate and depth over time have targeted muscle and/or metabolic fatigue as a key factor in performance deterioration[1] The work demand of CPR is described as modest to arduous, requiring between 60% and 65% of maximum achievable workload,[5,7] and as being a moderate aerobic exercise [4] requiring about 4 metabolic equivalents[8]. Individuals' subjective judgments evaluate CPR as very light work[9] and light to somewhat hard work[5]. Individuals who are physically fit can perform CPR in the training setting for longer periods of time than can those who are not fit,[10] but there are no differences in the compression rate or depth associated with fitness[8]. Blood lactate levels rise during CPR but those reported at the end of CPR[10] indicate that performance deterioration in CPR quality cannot be attributed to the accumulation of lactate and metabolic/muscle fatigue per se. None of the research on rescuer ability to maintain adequate rate and depth of chest compressions has specifically examined the recommended rescuer body position, the muscles activated to sustain the position, or the effect on the joints involved (wrist, shoulder, and hip).

### Purpose of the Study

The primary aim of this study was to compare rescuers' ability to deliver chest compressions of adequate rate and depth when performing Hands-Only and 30:2 CPR for 10 minutes. A secondary aim of the study was to evaluate fatigue levels and changes in body mechanics associated with doing each type of CPR. The specific questions that guided the study were

1. Are there differences in chest compression rate and depth associated with type of CPR (Hands-Only and 30:2)? Duration of CPR?

2. Are there differences in ability to deliver effective CPR (compression rate and depth) associated with age?

3. Are there differences in metabolic/muscular fatigue associated with type of CPR?

4. What changes in muscles and joints occur with CPR delivery? Are there differences associated with type of CPR?

## Methods

To evaluate rescuers' ability to deliver chest compressions of adequate rate and depth when performing Hands-Only CPR and 30:2 CPR, the study design captured select elements of the typical OOH resuscitation. In most OOH cardiac arrest situations, CPR is delivered with the victim lying on the ground. The rescuer kneels on the ground next to the victim and begins CPR. The most likely rescuer is a female 45 or more years old[11] Medical help arrives, on average, about 8 minutes after being called[12]

In this study, chest compressions were performed on a resuscitation manikin that was placed on the floor. The subject knelt on the floor next to the manikin to deliver CPR. Each subject delivered Hands-Only CPR and 30:2 CPR. All subjects were female. To examine the effects of age on quality of CPR, two age groups of subjects participated: half were between 22 to 35 years old and designated as younger while the other half were between 45 to 60 years old and designated as older. Each time rescuers delivered CPR, they were asked to do so for 10 minutes.

### Design

A three factor 2 × 2 × 2 (CPR type, age, and time) repeated measures cross-over design evaluated (1) chest compression rate, depth, and force (2) joint kinetics and kinematics during CPR, and (3) muscle activity while performing CPR. CPR type had two levels: Hands-Only and 30:2. Subjects performed both types of CPR with a mandatory 48 hours rest interval. Half of the subjects were randomized to do 30:2 CPR first and then Hands-Only CPR while the other half were randomized to Hands-Only CPR and then 30:2 CPR. Age had two levels: younger (22 to 35 years) and older (45 to 60 years). Time had two levels: early and end CPR administration. To standardize early CPR administration, the first 30 compressions of each trial were eliminated and the immediately following 90 compressions were considered as early CPR administration. End CPR was defined as the last 90 compressions of the CPR administered.

A 2 × 2 × 3 (CPR type × age × time) design evaluated metabolic/muscle fatigue as assessed by blood lactate level where time had three levels (pre-CPR, post-CPR, and 5 minutes post-CPR). A 2 × 2 × 2 (CPR type × age × time) design evaluated fatigue as assessed by ratings of perceived exertion where time had two levels (after 5 minutes of CPR and at end of CPR).

Data collection occurred in the University of Texas at Arlington Exercise Science Research Laboratory. The university's institutional review board reviewed and approved the study (IRB no. 07.122s).

### Subjects

The convenience sample for this study was recruited by flyers and word of mouth and consisted of 10 younger (age = 26 ± 3 years [M (mean) ± SD (standard deviation)]) and 10 older (age = 52 ± 4.5 years) healthy female volunteers with no prior or existing neuromuscular, musculoskeletal, or cardiopulmonary pathology and a body mass index (BMI) between 19 and 35 kilograms per meter squared (kg/m^2^). No other assessment of overall fitness was made.

The average BMI for younger subjects was 25 ± 5.6 kg/m^2 ^(range from 20.1 to 35.0 kg/m^2^) and 25 ± 3 kg/m^2 ^for older subjects (range from 19.8 to 30.2 kg/m^2^). Each held current AHA certification in basic life support for healthcare professionals. They were either graduate students or faculty in the university's school of nursing or department of kinesiology. None had performed CPR in response to a witnessed cardiac arrest. Each received compensation ($100/hour) for the time and effort associated with participation.

### Measures

Chest compression rate and depth were captured as each subject performed CPR on a Resusci Anne CPR Skillreporter (Laerdal Medical Corporation, Stavanger, Norway). A laptop computer connected to the Skillreporter continuously captured data on compressions using software provided by Laerdal Medical Corporation. The manikin was calibrated to provide data on depth of compressions that ranged between 1 and 55 mm. The force required to deflect the chest of the manikin at least 38 mm was 34.6 kilograms (kg). The average force needed to compress the chest of a normal patient to at least 38 mm is 32 kg. [13]

Fatigue was assessed in three ways: (1) the force with which chest compressions were delivered, (2) ratings of perceived exertion (RPEs), and (3) blood lactate level. To capture compression force data, the resuscitation manikin was placed on top of a force plate (AMTI model OR6-7-1000, Advanced Medical Technology, Watertown, MA). The top of the force plate was at the same level as the floor. Subjects self-assessed their level of fatigue using the Borg Rating of Perceived Exertion scale. Blood samples, obtained via finger prick, were analyzed using a YSI 1500 Sport Lactate Analyzer (YSI Incorporated, Yellow Springs, OH).

For neuromuscular assessment, surface electromyography (sEMG) captured data from the (1) lateral head of triceps brachii, (2) anterior deltoid, (3) pectoralis major, (4) biceps brachii, (5) lattisimus dorsi, (6) upper trapezius, (7) middle trapezius, (8) erector spinae, (9) external oblique, (10) hamstring, and (11) quadriceps. The sEMG sensors were single differential consisting of two parallel bars, each 1.0 centimeter (cm) long and 1 to 2 mm wide and spaced 1.0 cm apart, with a band width of 20 to 450 Hertz (Hz) and a roll-off of 80 decibels (dB)/decade. The sensors had a common mode rejection ratio of 92 dB, noise of 1.2 μV, and input impedance greater than 100 megaohms. The electrodes were placed on the midline of the muscle belly with the detection surface of the sensor perpendicular to the direction of the muscle fibers. The sEMG sensors were then connected to a 16-channel Bagnoli desktop EMG system (Delsys, Boston, MA). sEMG data from the 90 compressions at the start and the 90 compressions at the end of CPR were isolated. For each compression, the sEMG raw data were first rectified, the DC component removed, and a linear envelope obtained by low-pass filtering with a cut-off frequency of 20 Hz. The integrated EMG (IEMG) value was computed by integrating the linear envelope over each compression.

For biomechanical assessment of the joints, reflective markers were placed laterally on the left side of the subject on the neck at the level of 7^th ^cervical vertebra, the trunk at the level of 7^th ^thoracic vertebra, the trunk at the level of 4^th ^lumbar vertebra, the greater trochanter (hip), the lateral femoral epicondyle (knee), the lateral malleolus (ankle), the lateral edge of the acromion process (shoulder), the lateral humeral epicondyle (elbow), the ulnar styloid process (wrist); and, medially on the top of the head and the proximal phalanx of the third digit on the right hand. [14-16] A six-segment two-dimensional biomechanical model captured joint activity during CPR performance. The six segments included hands, forearms, upper arms, thighs, lower legs, and trunk. Segmental masses and center of mass locations were obtained from female anthropometric data tables. [14,16] A six- MCam2 camera Vicon 460 Motion Capture system (Los Angeles, CA) tracked the reflective markers to define the motion of each segment in the sagittal plane. A fourth-order low-pass Butterworth digital filter was used to smooth the video data with an 8-Hz cut-off frequency. A custom-built Visual Basic program (Microsoft Corporation, Redmond, WA) translated the digitized data and computed the angles, forces, and torques at the wrist, elbow, shoulder, and hip joint. Joint torque was defined such that positive torques caused extension of the joint and negative torques caused flexion of the joint.

### Procedures

As a subject was recruited, she was informed of the schedule for testing. She selected a date and time for her first of two testing occasions. Each occasion lasted no more than an hour and only one subject was tested on each occasion.

**Figure 1:**
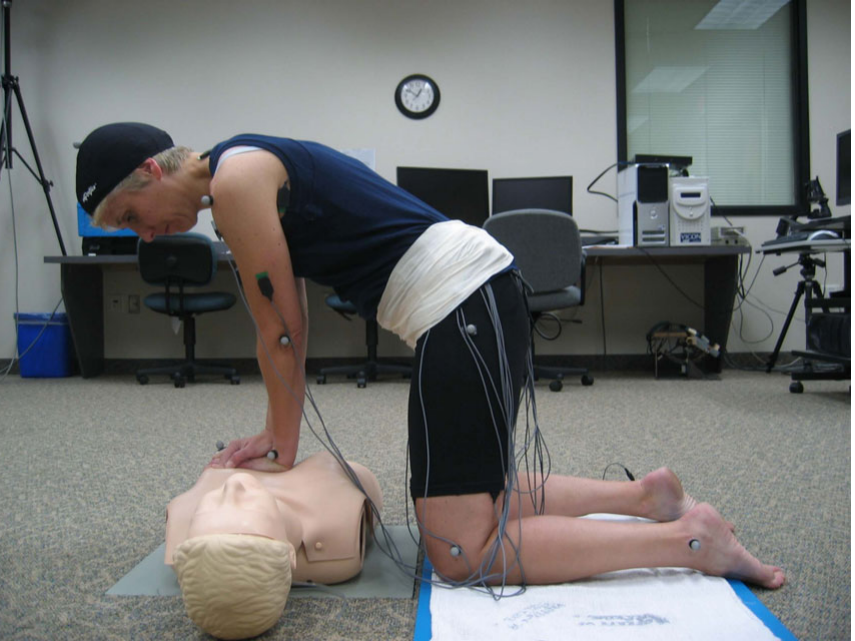
**Model depicting data collection during CPR**.

Upon arrival at the lab, the subject signed a consent form and completed a health questionnaire (used to ensure that she met the criteria for inclusion). Her height and weight were measured using a calibrated stadiometer and scale and then she donned spandex sports apparel to facilitate reflective marker and sEMG sensor placement (Figure 1).

After a blood sample was obtained, she was told to perform CPR until her rating of perceived exertion reached 17 or until she was told to stop. During the first 30 compressions only, she received active feedback about depth and rate of compressions and hand placement.

During the 10 minutes of CPR, compression rate, compression depth, video and force data were captured continuously. The force signals were obtained at a sampling frequency of 360 Hz, the video cameras sampled at a frame rate of 60 Hz, and the sEMG signals were sampled at 1000 Hz. At 5 minutes and again at the end of CPR, she provided a rating of perceived exertion (Borg scale). At the end of CPR and again 5 minutes after the end of CPR, a blood sample was obtained. At 5 minutes post CPR performance, she was asked if she experienced discomfort or pain in any joint; if she did, she identified the joint.

At the end of the first testing occasion, she scheduled a time for her second testing occasion (with a minimum of a 48 hour time lapse). On her second test occasion, the procedure for data collection was repeated as she performed the second type of CPR. At the end of the second test occasion, she received compensation for her time and effort.

### Statistical Analysis

NCSS 2001 (Salt Lake City, UT) was used to perform statistical analyses on the data. Each variable was evaluated for normality using Kolmogorov-Smirnov and Shapiro-Wilk normality tests. A three-way mixed factorial analysis of variance, comparing the age group effects (younger and older), CPR type effects (Hands-Only CPR and 30:2 CPR), and time effect (early CPR administration and end of CPR administration), was performed on the CPR performance data (rate of compressions, depth of compressions), the biomechanical (angles, torques, and forces for wrist, elbow, shoulder, and hip) and neuromuscular (IEMG and time of maximum activity of the muscle relative to the peak compression force) data, force of compressions, and ratings of perceived exertion. A three-way mixed factorial analysis of variance, comparing age group effects (younger and older), CPR type effects (Hands-Only CPR and 30:2 CPR) and time effects (pre-CPR, post-CPR and 5 minutes post-CPR), was used to examine blood lactate levels. All analyses of variance were interpreted using a step-down process beginning with the interactions of highest order. When the interactions were significant, simple effects were analyzed using Tukey-Kramer's post-hoc tests. For all the variables, the effect size and power of the statistic were estimated. The α level was set at .05.

## Results

All subjects completed the 10 minutes of 30:2 CPR; however, three subjects were unable to go beyond 5 to 6 minutes of Hands-Only CPR. One younger female (age = 24; mass = 54 kg; BMI = 20.4 kg/m^2^) completed 6 minutes of Hands-Only CPR; she performed Hands-Only CPR first and then 30:2 CPR. One younger female (age = 22; mass = 54.5 kg; BMI = 21.9 kg/m^2^) completed 5 minutes of Hands-Only CPR; she performed 30:2 CPR first and then Hands-Only CPR. One older female (age = 55; mass = 49 kg; BMI = 19.8 kg/m^2^) completed 6 minutes of Hands-Only CPR; she performed Hands-Only CPR followed by 30:2 CPR. Each reported physical exertion and joint pain as the reason to discontinue administering CPR.

### Chest Compression Rate and Depth

Across all subjects, average rate of compressions per minute tended to be under the recommended 100 per minute (Table 1) and average rate of compressions per minute was significantly lower (F(1,17) = 16.6, p < 0.001, η^2 ^= .97) during Hands-Only CPR (91 ± 2 number/minute) than during 30:2 CPR (98 ± 4 number/minute). There were no differences in rate of compressions associated with subjects' age group or duration of CPR (early and end CPR).

**Table 1:** Rate and depth of chest compressions (mean ± SE)

	Hands-Only CPR			30:2 CPR		
Measures	Early	End	Across time	Early	End	Across time
Compression rate (number per minute)*	90.4 ± 1.2	92 ± 3.5	91.2 ± 2.3	96.8 ± 0.5	98.9 ± 5.7	97.9 ± 3.5

Compression depth (mm)*	42.1 ± 2.7	36.3 ± 3.0	39.2 ± 4.1	43.7 ± 0.9	40.3 ± 2.5	42.0 ± 2.5
Percent of compressions 38 mm or more*	73.2 ± 10.5	46.6 ± 16.8	59.9 ± 19.1	82.3 ± 2.6	62.8 ± 16.4	72.5 ± 14.8

Hands-Only CPR and 30:2 CPR						

	Early		End		All Subjects (across time and CPR type)	

Compression rate (number per minute)	93.7 ± 0.8			95.5 ± 4.7		94.6 ± 1.8

Compression depth (mm)*	42.9 ± 1.8			38.3 ± 2.8		40.7 ± 2.3

Percent compressions 38 mm or more*	77.8 ± 4.0			54.7 ± 16.6		66.2 ± 10.4

Average depth of compressions (Table 1) was significantly lower (F(1,16) = 11.4, *p *< .004; η^2 ^= 0.89) for Hands-Only CPR (39 ± 4 mm) than it was for 30:2 CPR (42 ± 3 mm). In both types of CPR, average compression depth decreased significantly (F(1,16) = 27.7, *p *< .0001; η^2 ^= 0.99) from early (mean ± SE, 43 ± 2 mm) to end of CPR administration (38 ± 3 mm). There were no differences in average depth of compressions associated with subjects' age group.

The percentage of compressions that compressed the chest to at least 38 mm (Table 1) was significantly lower (F(1,16) = 4.5, p = 0.05, η^2 ^= 0.52) during Hands-Only CPR (60 ± 19%) than during 30:2 CPR (73 ± 15%). In both types of CPR, the percentage of compressions that compressed the chest to at least 38 mm declined significantly (F(1,16) = 14.6, p = 0.001, η^2 ^= 0.95) from early (78 ± 4%) to end CPR(55 ± 17%). There were no significant interactions and no differences associated with subjects' age group.

**Figure 2:**
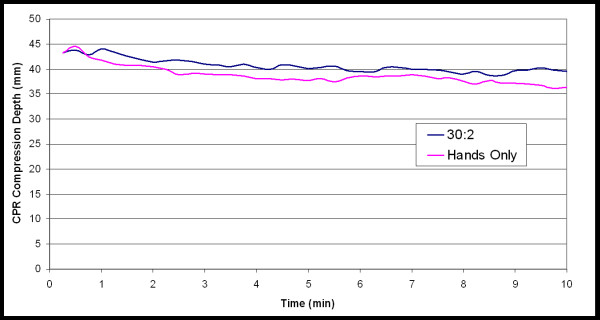
**Average compression depths across time for each type of CPR**.

**Figure 3:**
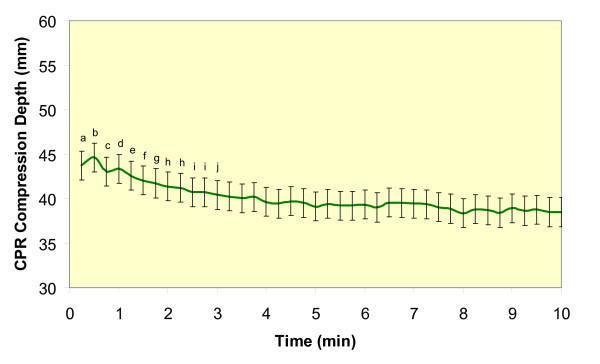
**Mean and standard error of compression depth when average at 15 second intervals**. a = 15 sec was different from 1 min 30 sec thru 10 min, b = 30 sec was different from 1 min 15 sec thru 10 min. c = 45 sec was different from 2 min 15 sec thru 10 min. d = 1 min was different from 2 min thru 10 min. e = 1 min 15 sec was different from 2 min 30 sec thru 10 min. f = 1 min 30 sec was different from 3 min thru 10 min. g = 1 min 45 sec was different from 3 min 15 sec thru 10 min. h = 2 min and 2 min 15 sec were different from 4 min thru 10 min. i = 2 min 30 sec and 2 min 45 sec were different from 5 min thru 10 min. j = 3 min was different from 7 min 45 sec.

To examine when in CPR administration and to what extent chest compression depth declined over time, depth of compressions for each 15-second interval was averaged. Average compression depth for Hands-Only CPR (39 ± 2 mm) was significantly lower (F(1,17) = 271.1, p < 0.0001, η^2 ^= 0.99) than it was for 30:2 CPR (41 ± 2 mm; Figure 2). There was also a significant time main effect for compression depth (F(39,733 = 10, p < 0.001, η^2 ^= 0.99; Figure 3). The post-hoc tests indicated that the following time points were significantly different: (a) 15 seconds was different from 1 minute 30 seconds through 10 minutes, (b) 30 seconds was different from 1 minute 15 seconds through 10 minutes, (c) 45 seconds was different from 2 minutes15 seconds through 10 minutes, (d) 1 minutes was different from 2 minutes through 10 minutes, (e) 1 minute 15 seconds was different from 2 minutes 30 seconds through 10 minutes, (f) 1 minute 30 seconds was different from 3 minutes through 10 minutes, (g) 1 minute 45 seconds was different from 3 minutes 15 seconds through 10 minutes, (h) 2 minutes and 2 minutes 15 seconds were different from 4 minutes through 10 minutes, (i) 2 minutes 30 seconds and 2 minutes 45 seconds were different from 5 minutes through 10 minutes, and (j) 3 minutes was different from 7 minutes 45 seconds through 10 minutes.

### Fatigue

A significantly greater decline in chest compression force occurred with Hands-Only CPR than with 30:2 CPR (F(1,14) = 7.12, p = 0.02, η^2 ^= 0.70; Table 2). Across all subjects, chest compression force declined significantly (F(1,14) = 38.97, p < 0.0001, η^2 ^= 0.99) from early (468 ± 6 Newton (N)) to end CPR(409 ± 6 N).

**Table 2:** Chest compression force (Newton; mean ± SE)

Hands-Only CPR			
	Younger	Older	All

Early	510.1 ± 7.6	416.2 ± 8.9	463.1 ± 5.7

End	440.0 ± 7.6	342.3 ± 8.9	391.1 ± 5.7

Across time	475.0 ± 14.0	379.3 ± 15.8	

30:2 CPR			

Early	498.1 ± 7.6	446.0 ± 9.3	472.1 ± 5.9

End	464.9 ± 7.6	390.1 ± 9.3	427.5 ± 5.9

Across time	481.5 ± 14.0	418.1 ± 17.1	

Hands-Only CPR and 30:2 CPR			

Early	504.1 ± 8.5	431.1 ± 10.0	467.6 ± 6.4

End	452.4 ± 8.5	366.2 ± 10.0	409.3 ± 6.4

Across time and type of CPR	478.3 ± 27.9	398.7 ± 32.8	

**Figure 4:**
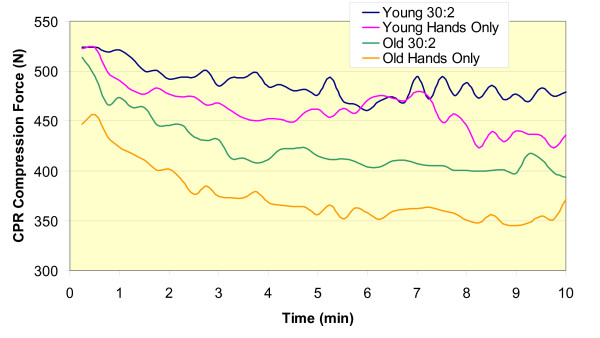
**Average compression forces across time for younger and older subjects performing Hands-Only and 30:2 CPR**.

To examine when in CPR administration and to what extent chest compression force declined, the peak vertical compression force in 15-second intervals throughout the duration of CPR administration for each type of CPR was averaged. The time × CPR type × age group and the age group × time interactions were not significant. There was a significant CPR × age group interaction (F(1,16) = 19.8, p = 0.0004; Figure 4). For younger subjects, mean compression force in Hands-Only CPR (458 ± 29 N) was significantly lower than in 30:2 CPR (488 ± 29 N). For older subjects, mean compression force in Hands-Only CPR (374 ± 33 N) was significantly lower than in 30:2 CPR (458 ± 29 N) and in 30:2 CPR younger subjects (488 ± 29 N) delivered significantly more forceful compressions than did older subjects (458 ± 29 N). Across both age groups, there was a significant difference in compression force associated with type of CPR (F(1,16) = 354.29, p < 0.0001). When collapsed across age groups, compression force was significantly lower in Hands-Only CPR (416 ± 22 N) than it was in 30:2 CPR (455 ± 22 N).

**Figure 5:**
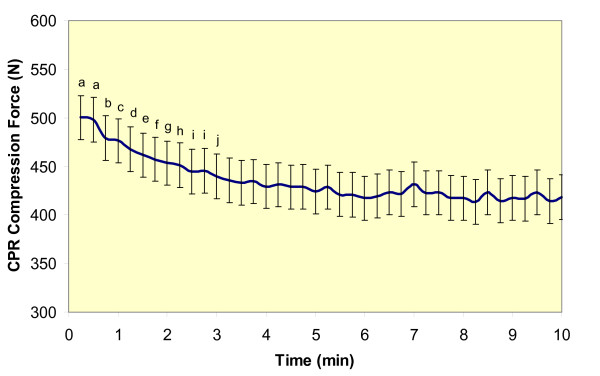
**Mean and standard error of compression force when averaged at 15 second intervals**. a = 15 & 30 sec were different from 45 sec thru 10 min. b = 45 sec was different from 1.5 min thru 10 min. c = 1 min was different from 1 min 45 sec thru 10 min. d = 1 min 15 sec was different from 2 min 30 sec thru 10 min. e = 1 min 30 sec was different from 3 min thru 10 min. f = 1 min 45 sec was different from 3 min 15 sec thru 10 min. g = 2 min was different from 3 min 15 sec thru 10 min. h = 2 min 15 sec was different from 3 min 30 sec thru 10 min. i = 2 min 30 sec and 2 min 45 sec were different from 5 thru 10 min. j = 3 min was different from 5 min 30 sec thru 10 min.

There was a significant time effect for chest compression force (F(1,39) = 12.27, *p *< .0001, η^2 ^= 0.999). Post-hoc analyses (Figure 5) revealed that the following time points were significantly different: (a) 15 and 30 seconds were different from 45 seconds through 10 minutes, (b) 45 seconds was different from 1.5 minutes through 10 minutes, (c) 1 minutes was different from 1 minute 45 seconds through 10 minutes, (d) 1 minute 15 seconds was different from 2 minutes 30 seconds through 10 minutes, (e) 1 minute 30 seconds was different from 3 minutes through 10 minutes, (f) 1 minute 45 seconds was different from 3 minutes 15 seconds through 10 minutes, (g) 2 minutes was different from 3 minutes 15 seconds through 10 minutes, (h) 2 minutes 15 seconds was different from 3 minutes 30 seconds through 10 minutes, (i) 2 minutes 30 seconds and 2 minutes 45 seconds were different from 5 through 10 minutes, and (j) 3 minutes was different from 5 minutes 30 seconds through 10 minutes.

Across all subjects, the ratings of perceived exertion (RPE) were significantly higher (F(1,18) = 19.12, *p *< .001, η^2 ^= 0.99) for Hands-Only CPR (14.5 ± 0.2) than they were for 30:2 CPR (13.3 ± 0.2; Table 3). Across both types of CPR, RPE values increased from 12.8 ± 0.2 at 5 minutes to 15.0 ± 0.2 at the end of CPR administration (F(1,18) = 85.56, p < .001, η^2 ^= 1.00).

**Table 3:** Ratings of perceived exertion on the Borg Scale (6 = very light and 17 = very hard; mean ± SE)

Hands-Only CPR			
	Younger	Older	All

5 minute	13.4 ± 0.2	13.4 ± 0.2	13.4 ± 0.2

End	15.5 ± 0.2	15.6 ± 0.2	15.5 ± 0.2

Across time	14.4 ± 0.3	14.5 ± 0.3	14.5 ± 0.2

30:2 CPR			

5 minute	12.3 ± 0.2	12.0 ± 0.2	12.2 ± 0.2

End	14.4 ± 0.2	16.7 ± 0.2	14.5 ± 0.2

Across time	13.4 ± 0.3	13.3 ± 0.3	13.3 ± 0.2

Hands-Only CPR and 30:2 CPR			

5 minute	12.8 ± 0.2	12.7 ± 0.2	12.8 ± 0.2

End	14.9 ± 0.2	15.1 ± 0.2	15.0 ± 0.2

Across time and type of CPR	13.9 ± 0.3	13.9 ± 0.3	13.8 ± 1.6

A significant time effect (F(2,34) = 49.82, *p *< .0000, η^2 ^= 1.00) was observed for blood lactate levels. Blood lactate levels were significantly higher post-CPR (2.7 ± 0.1 millimoles per liter (mM/L)) than they were at pre-CPR (1.3 ± 0.1 mM/L) and at 5 minutes post-CPR (2.1 ± 0.1 mM/L) when age and CPR type were collapsed (Table 4).

**Table 4:** Blood lactate levels (mM/L; mean ± SE)

Hands-Only CPR			
	Younger	Older	All

Pre	1.4 ± 0.2	1.5 ± 0.4	1.5 ± 0.1

Post	2.9 ± 0.3	3.1 ± 0.6	3.0 ± 0.1

5 minutes post	2.5 ± 0.4	1.9 ± 0.3	2.2 ± 0.4

30:2 CPR			

Pre	1.4 ± 0.2	1.0 ± 0.1	1.2 ± 0.3

Post	2.8 ± 0.3	2.1 ± 0.3	2.5 ± 0.5

5 minutes post	2.3 ± 0.2	1.6 ± 0.3	1.9 ± 0.5

Hands-Only CPR and 30:2 CPR			

Pre	1.2 ± 0.0	1.3 ± 0.4	1.3 ± 0.1

Post	2.9 ± 0.1	2.6 ± 0.7	2.7 ± 0.1

5 minutes post	2.4 ± 0.2	1.8 ± 0.2	2.1 ± 0.1

### Neuromuscular and Biomechanical Results

**Figure 6:**
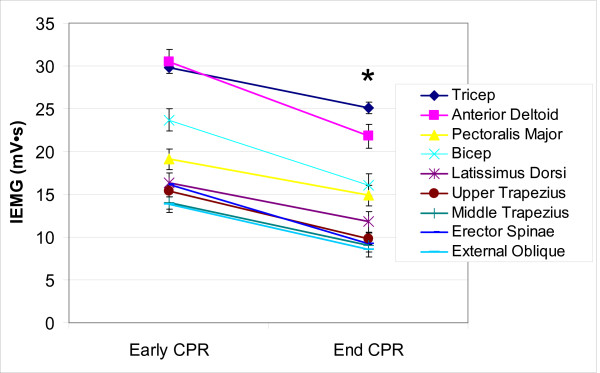
**Time effects for EMG Amplitude (IEMG)**. Bars represent Standard Error. * implies significant differences at p < 0.001. mV s is milivolts per second.

Muscle activity (IEMG amplitude) decreased significantly from early to end CPR in the anterior deltoid muscle, the pectoralis major muscle, the biceps brachii, the lattisimus dorsi muscle, the upper trapezius, the middle trapezius, and the external oblique muscles (Figure 6). Muscle activity in the major stalizing muscle groups (the lateral head of triceps brachii, erector spinae, hamstrings, and quadriceps muscles) did not change from early to end CPR.

Body segment angles (Table 5) did not change significantly from early to end CPR in either type of CPR. There were no significant changes in body segment angles associated with subjects' age group.

**Table 5:** Segment joint angles at peak compression (degrees, mean ± SE)

Forearm segment angles						
	Hands-Only CPR			30:2 CPR		

	Younger	Older	All	Younger	Older	All

Early	108.8 ± 0.7	112.6 ± 0.8	110.7 ± 0.6	109.2 ± 0.7	110.0 ± 0.8	109.8 ± 0.5

End	105.8 ± 0.7	110.5 ± 0.8	108.1 ± 0.6	107.1 ± 1.0	109.0 ± 0.8	108.1 ± 0.6

Across time	107.3 ± 2.2	111.5 ± 1.5		108.2 ± 0.2	109.5 ± 0.7	

Upper arm segment angles						

	Hands-Only CPR			30:2 CPR		

	Younger	Older	All	Younger	Older	All

Early	84.1 ± 1.6	83.1 ± 1.7	83.6 ± 1.2	83.2 ± 1.6	82.9 ± 1.6	83.1 ± 1.1

End	84.6 ± 1.6	83.9 ± 1.7	84.2 ± 1.2	80.6 ± 1.7	79.5 ± 1.6	80.0 ± 1.2

Across time	84.3 ± 1.3	83.5 ± 1.5		81.9 ± 1.4	81.1 ± 1.4	

Trunk segment angles						

	Hands-Only CPR			30:2 CPR		

	Younger	Older	All	Younger	Older	All

Early	214.3 ± 2.1	215.1 ± 2.4	214.7 ± 1.6	213.5 ± 2.1	215.9 ± 2.2	214.7 ± 1.5

End	214.1 ± 2.1	220.9 ± 2.4	217.5 ± 1.6	214.1 ± 2.7	215.0 ± 2.2	214.5 ± 1.6

Across time	214.2 ± 1.8	218.0 ± 2.1		213.8 ± 2.1	215.5 ± 1.9	

**Figure 7:**
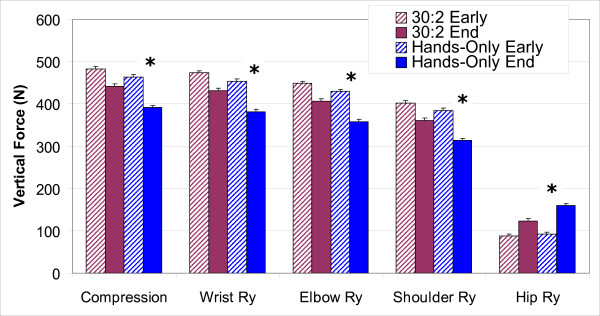
**Vertical joint forces: CPR type and time**. Bars represent Standard Error. * implies significant differences at p < 0.001. N is Newton.

Vertical joint reaction forces for the wrist (F(1,14) = 7.23, p = 0.02, η^2 ^= .71), elbow (F(1,14) = 7.27, p = 0.02, η^2 ^= 0.71), and shoulder (F(1,14) = 7.53, p = 0.02, η^2 ^= 0.72) showed a significantly greater decline over time with Hands-Only CPR than with 30:2 CPR (Figure 7). The vertical joint reaction forces for the hip (Figure 7) increased over time and were significantly greater (F(1,14) = 8.04, p = 0.02, η^2 ^= 0.75) in Hands-Only CPR (67 ± 5 N) than in 30:2 CPR (36 ± 5 N).

**Figure 8:**
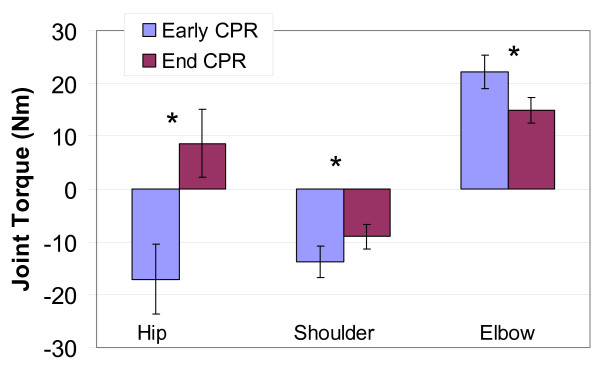
**Changes in joint torque from early to end CPR**. Bars represent Standard Error. * implies significant differences at p < 0.001. Nm is Newton metre.

Elbow extension torque and shoulder flexion torque declined significantly from early to end CPR in both types of CPR (F(1,14) = 21.79, *p *< .001, η^2 ^= .995 and F(1,14) = 10.33, *p *= .006, η^2 ^= .848). At the end of CPR, the shoulder and elbow were not as rigid as in early CPR; there was less torque in these joints at the end of CPR (Figure 8). In early CPR, the hip flexor muscles produced a flexion torque (positive values); however, at the end of CPR, the hip torque changed direction to produce hip extension (negative values; Figure 8). This change in hip torque was significant (F(1,14) = 39.88, p < .001, η^2 ^= .999).

All subjects reported pain/discomfort in one or more joints at 5 minutes after CPR performance. The most often identified joint, the one identified by each subject, was the wrist.

## Discussion

Hands-Only CPR required greater effort and was harder to sustain for ten minutes than was 30:2 CPR, as reflected in the objective and subjective data in this study. Younger and older subjects performed equally well in both types of CPR with similar changes in chest compression rate and depth associated with duration of CPR administration.

Average compression rate was somewhat less than optimal with both types of CPR and, as in reports of others, [4,5] remained consistent throughout the ten minute period. Compression rate during Hands-Only CPR was slower than for 30:2 CPR throughout the 10 minutes of CPR. Subjects' relative unfamiliarity with Hands-Only CPR may have contributed to the observed slower rate of chest compressions.

Differences in average depth of compressions over time associated with type of CPR, however, suggest that fatigue rather than unfamiliarity contribute to the shift from adequate to inadequate depth of compressions with Hand-Only CPR. Average depth of compressions with 30:2 CPR remained above 38 mm throughout the 10 minutes of CPR administration. Average depth of compressions with Hands-Only CPR dropped to and below 38 mm between 2 to 3 minutes; longer than the 1 minute reported by Ashton et. al[17] These findings are important as depth of compressions is a determinant of return of spontaneous circulation and 24-hour neurological outcomes in animal models of cardiac arrest [18-20] and increased defibrillation success[21] and short-term survival[22] in humans.

With both types of CPR, subjects experienced metabolic/muscular fatigue. The force with which subjects delivered chest compressions decreased significantly from early to end CPR and the decline was significantly greater with Hands-Only CPR than with 30:2 CPR. Subjects reported significantly greater levels of exertion when performing Hands-Only CPR than when performing 30:2 CPR. When doing 30:2 CPR they reported levels of exertion similar to that reported by others[23] Their ratings of perceived exertion indicate that they were working at a perceived effort of 65% (fairly light to somewhat hard) at 5 minutes, but by 10 minutes they were working at a perceived effort of 80% (hard). These results are comparable with that reported by Riera et al. who found that performing Hands-Only CPR for 2 minutes produced rescuer heart rates of 61% ± 8% (range, 49.5% to 75.5%) of theoretical maximum[7] Blood lactate levels support the subjective RPE data as there was a significant rise in blood lactate at the post-CPR measurement for both age groups and for both types of CPR. Before CPR, these subjects' lactate levels were within the 0.5–1.6 mM/L range observed during rest. The metabolic switch to anaerobic metabolism typically does not occur until blood lactate levels reach 4.0 mM/L[24] The post-CPR lactate levels for subjects in this study were well below this level and suggest that they were still using aerobic metabolism as their primary source of energy. These results are comparable with those reported by Baubin et al. where blood lactate was 2.9 ± 0.8 mM/L at the end of from 10 to 40 minutes of 5:1 dual-rescuer bag-valve-mask CPR[10] There were no differences in muscle activity (IEMG) associated with CPR type. The significant decreases in muscle activity illustrated in Figure 6 are most likely associated with a selective response to overall tiredness (RPE values) rather than true neuromuscular fatigue.

The biomechanical results of the study indicate that chest compression force during CPR is generated using gravity (free-fall) and hip flexion torque. The inertial force of gravity acting on the trunk is controlled by hip torque. Initially, subjects used a hip extension torque to hold the trunk up, which resists the inertial force of gravity. To compress the chest, subjects developed force by using gravity to accelerate the upper body downward. At peak chest compression, subjects increased the downward acceleration by producing trunk flexion torque. As subjects tired at the end of CPR administration, hip flexion torque switched to an extension torque. The decline in momentum resulting from this change in hip torque caused less force and torque to be transmitted down the arms. Changes in hip torque were accompanied by changes in elbow extension and shoulder flexion. At the end of CPR, both elbows and shoulders of these subjects were not as rigid as they were at the beginning of CPR. Both changes in hip torque and the significant reduction in IEMG amplitude of elbow and shoulder stabilizer muscles reduced the force transmitted down the arm contributing to decline in compression force and depth of chest compressions. Because subjects provided data about joint pain only at five minutes post-CPR, the role of wrist pain in compression force and depth decline remains unclear. Joint torque changes were more pronounced in Hands-Only CPR than in 30:2 CPR suggesting that Hands-Only CPR was more difficult to sustain than was 30:2 CPR.

The fine grain analyses of compression depth and force revealed similar patterns of decline over the course of the 10 minutes of CPR. Resuscitation training manikins, like the one used in this study, have spring loaded chests with a linear relationship between force and depth. In clinical settings just the opposite is true; there is a strong non-linear relationship between force and depth[13]. In adult OOH cardiac arrest patients, the force required to achieve chest deflections of 38 mm ranged from 10 to 54 kg (98 to 530 Newton), varied as a function of chest stiffness (stiffer chests were compressed more forcefully than were softer chests and softer chests were compressed more deeply than were stiffer chests), and declined significantly with the number of compressions performed. To achieve greater fidelity with clinical resuscitation requires resuscitation manikins that can simulate variation in chest stiffness as well as chest elasticity with increasing numbers of chest compressions. Until such manikins become available, the results of studies such as this one provide only a partial understanding of how well resuscitation training translates to clinical effectiveness.

During the first 30 compressions of each type of CPR, subjects in this study received feedback about the adequacy of their compressions (rate, depth and hand position). Ødegaard et al.,[25] Edelson et al[21] and Kramer-Johansen et al.,[22] among others, have reported that performance feedback devices used during 30:2 CPR mitigate the undesirable decrement in compression depth during professional resuscitation attempts with multiple rescuers. It would be of interest to know if feedback given to single rescuers delivering CPR over prolonged periods (especially Hands-Only CPR), as studied here, might also lead to improved performance. Further controlled laboratory study of how long and under what conditions feedback can help rescuers maintain compressions of minimum recommended depth is needed, and is the only practical way to assess this question as it relates to bystanders performing CPR for OOH cardiac arrest victims where feedback and data recording equipment would not likely be present.

In this study there were no differences in chest compression rate, chest compression depth, muscle activity, joint forces or perceived exertion associated with age group of the subject; however, the differential decline in force seen with older subjects suggests need for further examination of age associated differences. The effect sizes found in this study will be useful in identifying needed sample size in subsequent studies.

With each of the measures evaluated in this study (except rate), changes from early to end CPR were greater in Hands-Only CPR than they were in 30:2 CPR. It is not certain if the additional decrement of chest compression depth observed with Hands-Only CPR is of clinical significance. A previous animal study using a canine model of cardiac arrest suggests that relatively small incremental changes in compression depth during CPR could yield a significant reduction in cardiac output and mean arterial blood pressure and that the relationship is linear above some threshold compression depth[20] In a human trial, Edelson et al. [21] showed that a 5 mm increase in the mean depth of compressions just prior to a defibrillation attempt could approximately double the chance of cardioversion. The additional decrement in mean compression depth associated with prolonged performance of Hands-Only CPR in this study was only 2.8 mm; but, the percentage of compressions with depths of at least 38 mm (the minimal recommended depth in adult victims) delivered during Hands-Only CPR was significantly less than those delivered during 30:2 CPR. If it is assumed that those compressions less than 38 mm in depth would not affect optimal perfusion during an actual resuscitation, the impact of the reduced number of adequate compressions during Hands-Only CPR could offset some of the benefit of not pausing for ventilations when using 30:2 CPR.

The data observed in this study support the assumption that the pauses during compressions that are required in 30:2 CPR for ventilations provide 'rest time' for the muscles that are used to perform compressions and therefore contribute to a lower level of fatigue than when doing Hands-Only CPR. Like the use of feedback, then, perhaps short rest periods may mitigate the decrement in compression depth that was seen to be associated with Hands-Only CPR. Further study of the impact of brief rest periods on fatigue during prolonged Hands-Only CPR may provide guidance on the minimal duration and frequency of rest periods required to maintain optimal performance of chest compressions when doing Hands-Only CPR. Coupled with physiological data from actual resuscitations, such experimental performance data should allow further refinement of CPR guidelines and training.

## Conclusion

Hands-Only CPR for adult victims of OOH witnessed sudden cardiac arrest is a simpler procedure to learn than 30:2 (traditional) CPR because it requires no interruptions to compressions for ventilations. This study shows that Hands-Only CPR, when performed on a resuscitation manikin, does require more effort, over time, than does 30:2 CPR and is harder to sustain. Mean compression depth declines sooner and fewer compressions greater than the recommended minimum depth are delivered with Hands-Only CPR than with 30:2 CPR. Further examination of ways to optimize performance of Hands-Only CPR, perhaps through use of feedback devices and by reducing fatigue with brief rest periods, is crucial to improving CPR guidelines and training and increasing the likelihood that bystanders of witnessed OOH cardiac arrest will perform lifesaving resuscitation.

## Competing interests

JP is employed by the American Heart Association. The American Heart Association produces and markets CPR training materials. WCP is employed by Laerdal Medical Corporation. Laerdal Medical Corporation manufactures and markets training manikins including those used in CPR training. CLC hold research grants from Laerdal Medical Corporation for other projects and serves as a for-fee consultant to the American Heart Association. All other authors declare that they have no competing interests.

## Authors' contributions

CT participated in all aspects of the project. JNP managed all data collection and assisted with data analyses. MDR participated in all aspects of the project. JP helped interpret the results and draft the manuscript. WCP helped design the study and commented on the results. CLC secured funding for the project and participated in all aspects of the project. All authors read and approved the final manuscript.

## Pre-publication history

The pre-publication history for this paper can be accessed here:


